# Press needle for aspiration pneumonia prevention in older adults: Study protocol for a randomized double-blind placebo-controlled trial

**DOI:** 10.1097/MD.0000000000032847

**Published:** 2023-02-17

**Authors:** Soichiro Kaneko, Akiko Kikuchi, Shin Takayama, Ryutaro Arita, Minoru Ohsawa, Tetsuharu Kamiya, Tadashi Ishii

**Affiliations:** a Department of General Practitioner Development, Tohoku University Graduate School of Medicine, Sendai, Japan; b Department of Kampo and Integrative Medicine, Tohoku University Graduate School of Medicine, Sendai, Japan; c Department of Education and Support for Regional Medicine, Department of Kampo Medicine, Tohoku University Hospital, Sendai, Japan.

**Keywords:** aspiration pneumonia, older adults, press needle, randomized double-blind placebo-controlled trial

## Abstract

Pneumonia is the fifth most common cause of death among the Japanese population, with 97% of the deaths occurring among older adults aged ≥65 years. The incidence ratio of aspiration pneumonia is high among the older adults. Therefore, its prophylaxis is important in geriatric medicine. In our previous studies, we reported that stimulation of acupoints at stomach meridian 36 and kidney meridian 3 of the lower limbs with a press needle improved the swallowing function of patients with dysphagia. Improvements in swallowing function may prevent aspiration pneumonia. This study aims to investigate the protective efficacy of press needle stimulation in the lower limbs for aspiration pneumonia.

**Methods/design:**

This is a multicenter, randomized, double-blind, placebo-controlled trial. A total of 140 patients with cerebrovascular disorder and a history of aspiration pneumonia will be recruited from 6 centers and randomly assigned to either the real or sham press needle group in a 1:1 ratio. The press needle will be replaced twice a week. The treatment will be administered bilaterally at acupoints stomach meridian 36 and kidney meridian 3. The primary outcome is the frequency of aspiration pneumonia onset. The secondary outcome is the improvement of the latent time of the swallowing reflex. The study period is of 12-month. The primary outcome will be evaluated throughout the study period, while the secondary outcomes will be assessed at baseline, 1st month, 6th month, and at the end of the investigation period.

**Discussion:**

This study will evaluate the effects of press needle on the prevention of aspiration pneumonia and the improvement of swallowing function in patients. The results of this study will help support the prophylaxis of aspiration pneumonia.

## 1. Introduction (6a, 6b, 7, 8)

In Japan, pneumonia is the fifth most common cause of death worldwide. Moreover, 97% of the deaths due to pneumonia occur among older adults aged ≥65 years. In addition, the ratio of aspiration pneumonia to age-specific pneumonia increases with aging.^[1]^ Among patients with pneumonia aged ≥70 years, approximately 70% are considered to have aspiration pneumonia.^[2]^ Particularly, patients with cerebrovascular disorders are easily affected by aspiration pneumonia.^[3,4]^ In other words, aspiration pneumonitis is an important disease associated with vital prognoses in older adults. Aspiration pneumonia is caused by an influx of saliva and other fluids, particularly at night. A decrease in the swallowing reflex is strongly related to the onset of aspiration pneumonia.^[5]^ Furthermore, physiological changes associated with aging contribute to this decrease.^[6]^ The causes include a decrease in masticatory force due to missing teeth, poor alimentary bolus formation with decreased capacity of saliva secretion, delay in swallowing reflex creation with a decrease in pharyngeal perception, delay of the elevation by the low degree of the laryngeal prominence, and parorexia with a decrease in cognitive function.^[7,8]^ In addition, a history of cerebrovascular disorders results in a decrease in substance P, an important trigger for the swallowing reflex, and reduces swallowing function.^[9]^

Strategies for preventing aspiration pneumonia include improvement of swallowing function with rehabilitation, pharmacotherapy mouth care, prevention of gastroesophageal reflux, maintenance of consciousness, maintenance of nutritional status, and vaccination against pneumococcus.^[10]^ However, the number of patients with aspiration pneumonia has increased.^[1]^ Therefore, novel therapies that improve swallowing function and prevent aspiration pneumonia are required.

**Figure 1.**
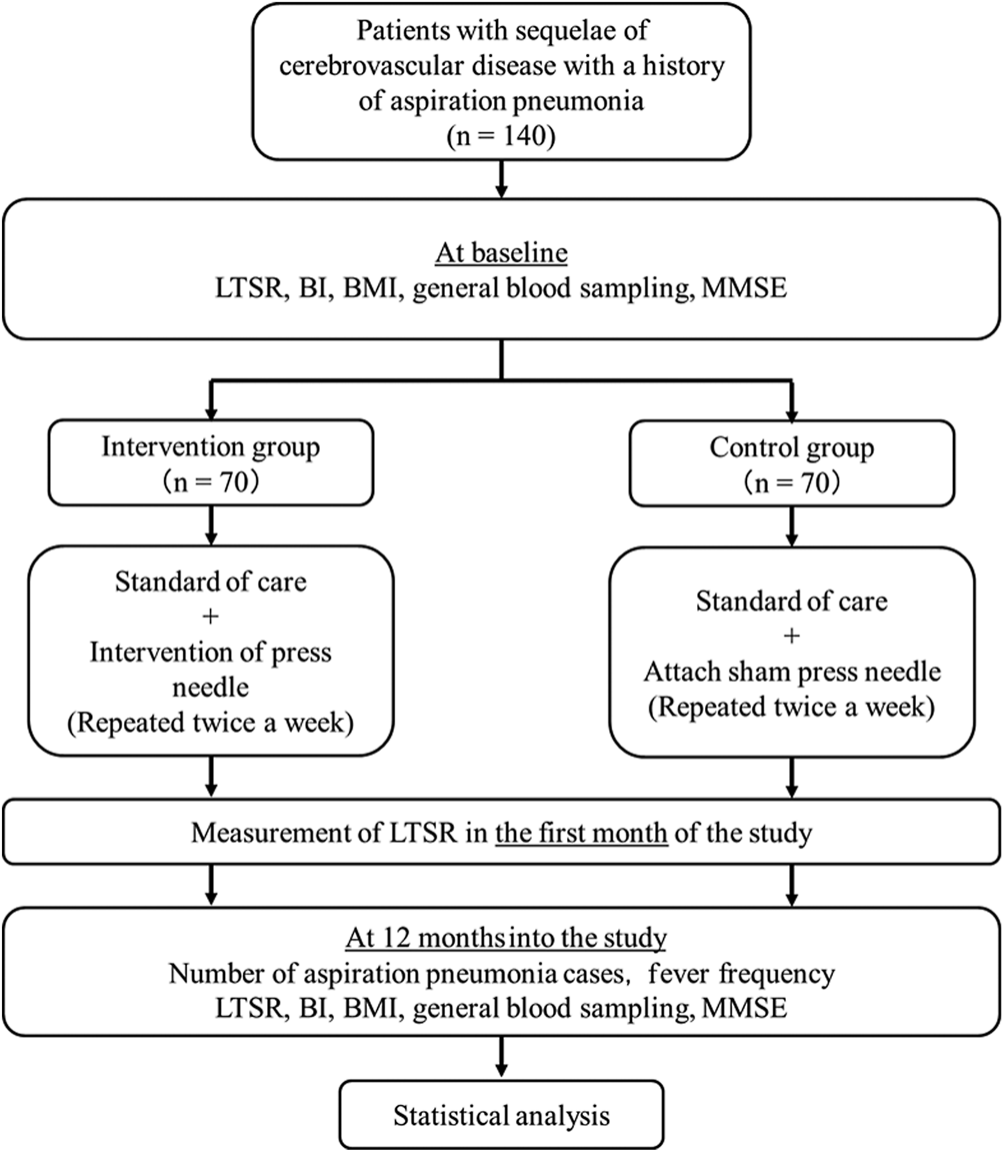
Flow chart of the clinical trial. LTSR = latent time of swallowing reflex, BI = Barthel index, BMI = body mass index, LTSR = latent time of swallowing reflex, MMSE = mini-mental state examination.

We previously reported improvements in swallowing function in older adults with cerebrovascular disorders using acupuncture stimulation.^[11–13]^ However, the protective efficacy of acupuncture stimulation against aspiration pneumonia has not yet been reported. This study aimed to investigate the protective efficacy of press needle stimulation in the lower limbs for aspiration pneumonia. This study will be a randomized double-blind placebo-controlled trial to assess the efficacy and safety of press needles for aspiration pneumonia and swallowing function in patients with cerebrovascular disease and a history of aspiration pneumonia. Participants will be blinded to the treatment assignment throughout the trial. The study design is illustrated in Figure 1.

## 2. Methods/design

### 2.1. Study setting (9)

Potential participants with cerebrovascular disease with a history of aspiration pneumonia will be recruited from the National Hospital Organization Yonezawa Hospital, Minamisanriu Hospital, Kesennuma City Motoyoshi Hospital, Saka General Hospital, Sendai Tomizawa Hospital, and Ikeno Clinic in Japan. The list of the institution is submitted to the Tohoku University clinical research Ethical Review Board and can obtain it from there.

### 2.2. Eligibility (10)

Older patients who met all of the following inclusion criteria and do not meet any of the listed exclusion criteria will be eligible to participate:

#### 2.2.1. Inclusion criteria.

1. Patients with cerebrovascular disease and a history of aspiration pneumonia.
2. Patients in the life stage (maintenance or chronic stage) of rehabilitation for cerebrovascular disease.
3. Patients of a joint research facility receiving in patient or home care.

#### 2.2.2. Exclusion criteria.

1. Patients with serious diseases, such as chronic respiratory diseases, malignancies, and chronic ischemic diseases, that can result in pneumonia.
2. Patients with metal allergies.
3. Patients with severe skin disorders.
4. Patients with no developed swallowing reflex at the time of the initial latent time of swallowing reflex (LTSR) measurement.
5. Patients with 3 or more severe aspiration pneumonia episodes in the past 3 months.

Patients receiving the standard treatment for cerebrovascular disease, its sequelae, and aspiration pneumonia are acceptable. However, the use of high-dose steroids and immunosuppressive therapies are unacceptable.

### 2.3. Interventions (11)

#### 2.3.1. Intervention description {11a}.

**Figure 2.**
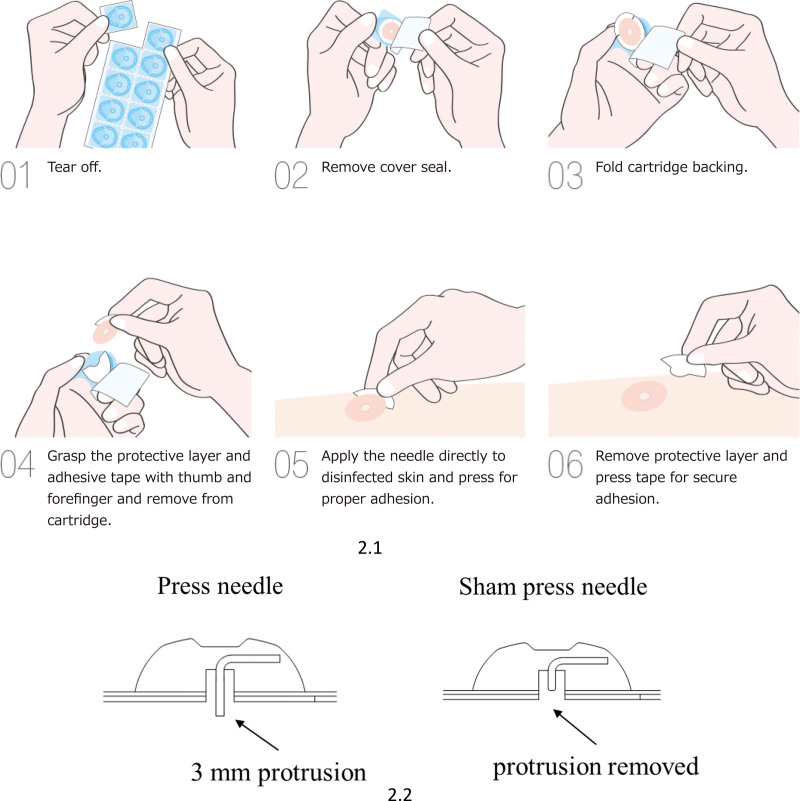
1. Way of applying Pionex Zero.^[15]^ The press needle used in this study is a Pionex Zero (Seirin Corporation, Shizuoka, Japan). The patented plastic case and sheath allow the practitioner to insert the needle without touching the needle tip or the surface of the Micropore™ adhesive tape. 2. Images of the press and sham press needle used in this study. The press needle has a non-sharp protrusion of 3 mm. In the sham press needle the protrusion is removed.

Participants will receive press needle stimulation at 2 acupuncture points, ST36 (Zusanli) and kidney meridian 3 (Taixi),^[14]^ on the lower limbs for 12 months. According to traditional Chinese medicine, stimulation of these 2 acupuncture points is beneficial for age-related decline in swallowing function. A press needle is a therapeutic device in which an acupuncture needle is safely secured using a circular surgical tape. Pionex Zero (Seirin Corporation, Shizuoka, Japan) noninvasive press needles will be used in this study (Fig. 2). The irritation area in contact with the skin is not a needle but a protrusion that is designed to not penetrate the skin. While the previous study used an invasive device,^[11]^ the present study used a safer device, considering the observation period of 12 months. The safety and quality of Pionex Zero are controlled by the Japanese Ministry of Health, Labour, and Welfare (Notification Number:22B1X00006000004). As a sham press needle was used for the control group, the protruding part of the Pionex Zero will be removed. The authenticity of the sham device has been confirmed.^[16]^ The press and sham press needles will be replaced twice weekly in the intervention and control groups.

#### 2.3.2. Criteria for discontinuing or modifying allocated interventions {11b}.

In cases of skin abnormality in the area of application of the press needles, physicians will be consulted and the intervention paused for a period of time. Subsequently, physicians will be consulted about the possibility of resuming treatment. The intervention will also be discontinued if other diseases develop during the period and require treatment.

#### 2.3.3. Strategies to improve adherence to interventions {11c}.

Frequent bedside visits and health assessments will be an important part of monitoring adherence.

#### 2.3.4. Relevant concomitant care permitted or prohibited during the trial {11d}.

Usual treatments for cerebrovascular disease, its sequelae, and aspiration pneumonia are acceptable. High-dose steroids and immunosuppressive therapy are unacceptable.

### 2.4. Outcomes (12)

The primary outcomes will help determine whether aspiration pneumonia is prevented and if press needle stimulation is effective. The primary outcome is the number of cases of aspiration pneumonia during the study period. Diagnosis of aspiration pneumonia will be made by the attending physician based on the Japanese Respiratory Society Guidelines for the management of hospital-acquired pneumonia in adults. The total number of diagnoses of aspiration pneumonia at the end of the study will be calculated.

Secondary outcomes will help determine whether seal acupuncture improves swallowing function and general conditions. The secondary outcomes are LTSR, fever frequency, Barthel index (BI), body mass index, general blood test results, and mini-mental state examination (MMSE) results. LTSR measures the time between the insertion of an 8 Fr tube through the nasal cavity to the uvula of the palate and the initiation of swallowing after the injection of 1 mL of distilled water. Three measurements are taken and the mean value is calculated. Fever is defined as a body temperature of 37.5°C or more. The number of days with fever during the study period will be determined.

### 2.5. Participant timeline (13)

The time schedule for this trial and data collection is summarized in Figure 2.

### 2.6. Sample size (14)

The sample size calculation is based on the results of previous studies. The incidence of aspiration pneumonia in previous studies was 0.091 (4/44) in the intervention group and 0.292 (14/48) in the control group.^[17]^ The sample size has been calculated with a 2-tailed significance level of 5% and a power of 80%, resulting in 118 participants (59 in each group). To account for 20 dropouts, the calculation 118 + 20 = 138 suggests that 138 patients should be recruited. This value has been rounded off to 140 patients.

### 2.7. Recruitment (15)

Potential participants with cerebrovascular disease and a history of aspiration pneumonia will be recruited from the National Hospital Organization Yonezawa Hospital, Minamisanriu Hospital, Kesennuma City Motoyoshi Hospital, Saka General Hospital, Sendai Tomizawa Hospital, and the Ikeno Clinic. The physician will contact the administrator when the patient meets the eligibility criteria. If participants are interested in our study, they will be informed of the purpose and content of the research as well as the benefits and drawbacks of participating in detail. After screening, the participants who meet the inclusion criteria will be enrolled. The study flow is shown in the SPIRIT Figure (Fig. 1). The participants will undergo a 12 months treatment period.

### 2.8. Randomization, allocation, and blinding (16)

#### 2.8.1. Method of generating the allocation sequence {16a}.

**Figure 3.**
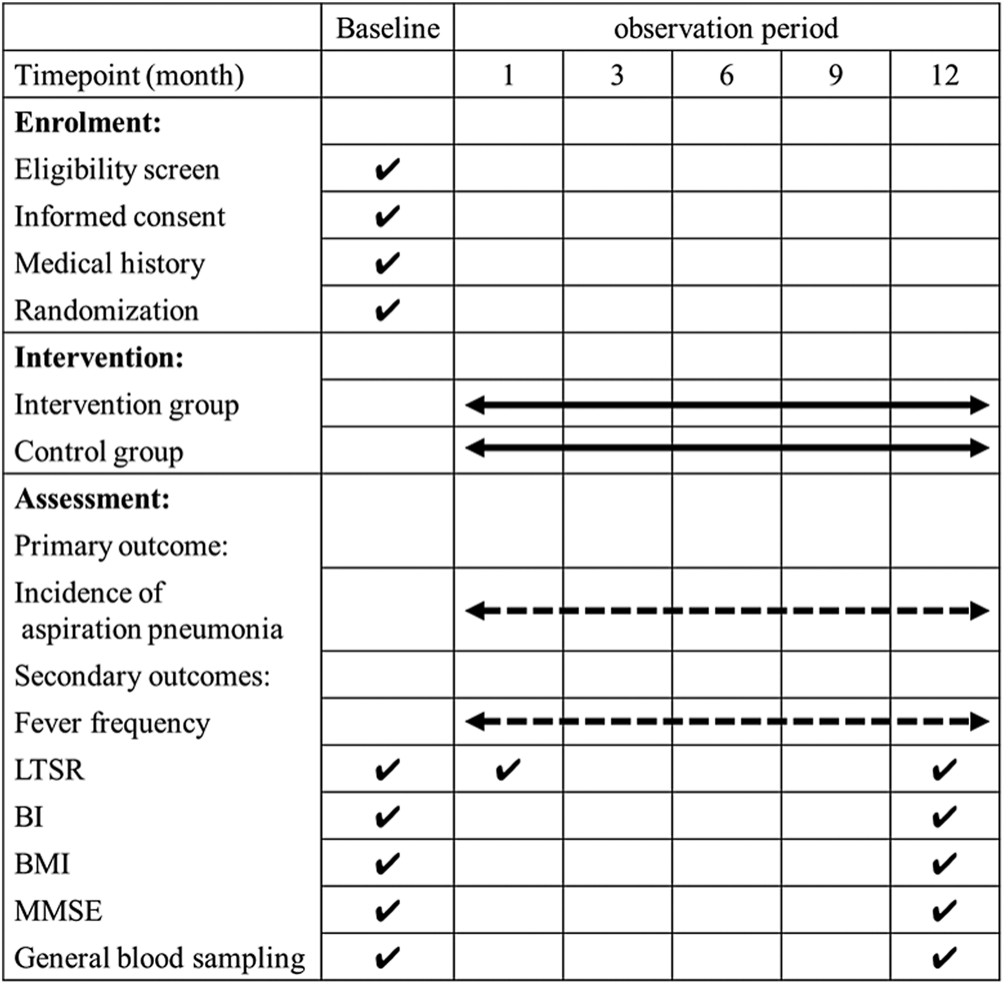
Measurement items and data collection plan. The number of occurrences of aspiration pneumonia and frequency of fever during the 12 months observation period will be determined. LTSR = latent time of swallowing reflex, BI = Barthel index, BMI = body mass index, LTSR = latent time of swallowing reflex, MMSE = mini-mental state examination.

After obtaining written informed consent from the participants and baseline screening. Random numbers of 001 to 140 will be automatically generated by the SPSS software (whole random numbers). The participants will be randomly assigned to the intervention group or control group at a ratio of 1:1. An overview of the case distribution of each center, specific measurements, and time points of data collection can be found in the SPIRIT Figure (Fig. 3).

#### 2.8.2. Mechanism of implementing the allocation sequence {16b}.

The random allocation sequence will be stored and concealed from the treating physicians, statisticians, and outcome assessors to prevent detection bias.

#### 2.8.3. Who will generate the allocation sequence{16c}.

The designated statisticians will generate the allocation sequence, and different investigators designated by the project leader will enroll or assign participants.

#### 2.8.4. Who will be blinded {17a}.

Information including the number of cases of aspiration pneumonia, LTSR, fever frequency, BI, body mass index, general blood sampling, and the MMSE will be assessed by independent assessors who are blinded to the assignment and treatment. The principal investigators, statisticians, and outcome assessors will be blinded to the treatment assignments until the database is locked.

#### 2.8.5. Procedure for unblinding if needed {17b}.

Unblinding the investigators will be permissible only in specific situations, such as when knowledge of the actual treatment is highly necessary for the appropriate management of participants (e.g., serious adverse events).

### 2.9. Data collection (18a, 18b)

An overview of specific measurements and time points for data collection is shown in the SPIRIT Figure (Fig. 3). All data will be collected and recorded on paper-based case report form (CRF) by the investigators. Patient characteristics, questionnaires, and evaluation forms, such as BI and MMSE, findings of LTSR swallowing measurement and number of incidences of aspiration pneumonia, fever frequency, laboratory data, and physical status will be collected. Adverse effects will also be recorded. After recording the data, the investigators will confirm the CRF form by referring to the medical records.

### 2.10. Data management (19)

We will assign identification code numbers to all participants. Upon registering and recording the CRF, personal identifying information (e.g., name, address, phone number, and medical record number) will be deleted from the data. A linkable anonymized number table will be created and stored by the data manager. All data will be kept in locked storage for at least 5 years after study completion. The data manager will confirm the CRF to check for duplication, missing information, and deviation from the data.

### 2.11. Statistical methods (20a, 20b, 20c)

#### 2.11.1. Statistical methods for primary and secondary outcomes (20a).

Statisticians will independently undertake statistical analyses. Parametric data will be presented as mean ± standard deviation, whereas non-parametric data as median and 95% confidence interval. Two-tailed *t* tests and chi-squared tests will be used to compare the demographic and clinical characteristics of the 2 groups at baseline. MMSE, BI, and total caloric intake at baseline and endpoint will be compared between the 2 groups using repeated-measures analysis of variance. The number of febrile days in the 2 groups was compared using the 2-tailed *t* test. The probabilities of being pneumonia-free and survival from pneumonia-related deaths will be estimated using the Kaplan–Meier product-limit method. Pneumonia-free survival rates will be calculated from the date of random assignment to the date of pneumonia onset, date of death, or cutoff date for patients alive at the time of closure of the dataset. A Cox proportional hazards regression model will be used to examine the relationship between modifiable factors and the incidence of pneumonia or pneumonia-related mortality. Relative risks) and 95% confidence interval will be calculated from 2-by-2 tables. Plausible predictors (age, sex, ease of diagnosis, duration of illness, activities of daily living, cognitive function, and treatment assignment) will be included in the original model. Backward stepwise regression will be performed, and *P* > .2 will be used for variable removal. Statistical analysis will be performed using SPSS software (version 21.0; IBM, Armonk, NY). All statistical tests will be 2-sided, and the statistical significance threshold will be set at 5%.

### 2.12. Data monitoring

#### 2.12.1. Composition of the data monitoring committee, its role and reporting structure (21a). 2.12.

The principal investigator will ask the data controller and monitoring personnel to monitor the study to ensure that it is being conducted safely and in accordance with the research protocol with accurate data collection.

The principal investigator will prepare a monitoring plan, and the data controller and monitoring staff will conduct monitoring in accordance with the monitoring plan.

### 2.13. Harms

#### 2.13.1. Adverse event reporting and harms (22).

Adverse events, such as signs, symptoms, and other discomfort, will be observed and recorded in detail during the study. The attending physician will take appropriate action in the event of a serious adverse event or failure and report it to the principal investigator of the institution. The principal investigator will review information regarding the type of adverse event, the severity classification, the severity and reason for the determination of severity, predictability, the causal relationship with the intervention, the history of the event, and the participant identification. Severity classification will be determined according to the National Cancer Institute Common Terminology Criteria for Adverse Events (NCI CTCAE v4.0: http://www.jcog.jp/doctor/tool/CTCAEv4J_20150310.pdf).

### 2.14. Auditing (23)

#### 2.14.1. Composition of the coordinating center and trial steering committee {5d}.

The investigators will prepare a written audit plan and based on this, visit the institution to check the approval documents by the head of the institution, review the explanatory and consent documents, and check the contents of the CRFs against medical records. The results of the audit will be submitted to the principal investigator, principal investigator of the institution, and head of the institution.

### 2.15. Ethics and dissemination

#### 2.15.1. Ethics approval and consent to participate (24).

This trial complied with the Declaration of Helsinki and JP Clinical Trial Act.^[18]^ The research ethics committee of Tohoku University approved this trial protocol.

#### 2.15.2. Plans for communicating important protocol amendments to relevant parties (e.g., trial participants, ethical committees) {25}.

If there are changes to the eligibility criteria, outcomes, and analyses, a new version of the protocol will be submitted to the Tohoku University Hospital Ethics Committee for approval.

##### 2.15.2.1. Who will take informed consent? {26a}.

The study administrator or a physician at the collaborating hospital will explain the procedure to the patient or family and obtain their informed consent.

##### 2.15.2.2. Additional consent provisions for collection and use of participant data and biological specimens {26b}.

Not applicable.

#### 2.15.3. Confidentiality {27}.

Participants personal information will be kept confidential in the same way as their medical records in the hospital before, during, and after the trial.

#### 2.15.4. Conflict of interest (28).

The authors declare that they have no competing interests.

### 2.16. Trial status

The protocol and progress are registered in the University Hospital Medical Information Network (UMIN000023123). Recruitment will begin in January 2022 and is expected to be completed in May 2025.

### 2.17. Funding (4)

This study is supported by Grants-in-Aid for Scientific Research (KAKENHI) grant number 15K08897 (Japan).

## 3. Discussion

This study investigated the aspiration pneumonitis prevention of patients with cerebrovascular disorder using practical acupuncture stimulation for the first time. Aspiration pneumonia is primarily caused by subclinical aspiration of nasal, laryngeal, and periodontal secretions that go unnoticed mainly at night. One of the mechanisms by which aspiration occurs is through decreased swallowing and coughing reflexes. The proper functioning of these reflexes requires that substance P synthesized in the cervical ganglion, the sensory branch of the vagus and glossopharyngeal nerves, be maintained at a constant concentration in the peripheral nerves of the pharynx and endotracheal tissue. Substance P is also enhanced by dopamine, which is synthesized in the substantia nigra and striatum. When the deep cortex, including the substantia nigra and striatum, is impaired due to cerebrovascular disease, the ability to synthesize dopamine is also impaired, leading to a decrease in swallowing function.^[9]^ Acupuncture stimulation of the neck improves swallowing function and the expression of 5-HT_1A_ in the arcuate nucleus.^[19]^ Studies that directly investigate the mechanism of dopamine synthesis in the nigrostriatal striatum, as described above, are lacking. However, the use of acupuncture for the treatment of cerebrovascular disease^[20]^ has been shown to improve cerebral blood flow in aged rats and reduce infarct volume.^[21]^ Studies using rats with Parkinson disease, which is a different disease with similar striatum nigra involvement, have shown that acupuncture prevents cell death in the nigra and striatum.^[22]^ The addition of acupuncture to the treatment of patients with Parkinson disease has been reported to significantly increase blood dopamine.^[23]^ From these reports, it can be inferred that acupuncture stimulation may promote dopamine synthesis in the substantia nigra striata as a probable mechanism for improving swallowing function.

In this study, the stimulation will be delivered to the lower limb. This study is based on previous studies showing that stimulation of the lower limbs of patients with cerebrovascular disease improved swallowing function.^[11]^ However, stimulation sites other than the lower limbs have been reported to improve swallowing dysfunction.^[19]^ In the present study, the lower limb was chosen as the stimulus site because of the possibility of continuing stimulation during the 12 months observation period.

The press needles and placebo press needles that will be used in the real stimulation and the control group, respectively are identical in appearances when attached to the skin. Furthermore, there is no difference between the 2 devices in terms of the sensations felt by the participants. Thus, it will be difficult for participants to distinguish between the 2 devices.^[16]^

The same investigator will be responsible for the examination of each patient at different time periods.

All indicators will be assessed by independent assessors.

The principal investigators, statisticians, and outcome assessors will be blinded to treatment assignments until the database is locked.

In conclusion, this study aims to test the therapeutic efficacy and safety of acupuncture stimulation for the prevention of aspiration pneumonia in patients with cerebrovascular disease.

The results of this study will provide evidence-based data, thus opening a new avenue for the use of acupuncture stimulation for aspiration pneumonia prophylaxis in patients with cerebrovascular disease.

## Acknowledgments

We would like to thank Ms. Akiko Kuwabara for her assistance as clinical research coordinator. The authors would like to express their appreciation for the efforts of all researchers in this trial. We would like to thank Editage (www.editage.com) for the English language editing.

## Author contributions

**Conceptualization:** Shin Takayama, Tadashi Ishii.

**Data curation:** Tetsuharu Kamiya.

**Formal analysis:** Soichiro Kaneko, Ryutaro Arita.

**Funding acquisition:** Soichiro Kaneko.

**Investigation**: Akiko Kikuchi, Shin Takayama, Minoru Ohsawa, Tetsuharu Kamiya.

**Methodology**: Soichiro Kaneko, Ryutaro Arita, Akiko Kikuchi, Shin Takayama.

**Project administration**: Shin Takayama, Tadashi Ishii.

**Resources**: Shin Takayama, Tadashi Ishii.

**Supervision:** Akiko Kikuchi, Ryutaro Arita, Shin Takayama, Tadashi Ishii.

**Visualization**: Soichiro Kaneko.

**Writing – original draft**: Soichiro Kaneko.

**Writing – review & editing**: Akiko Kikuchi, Shin Takayama, Ryutaro Arita, Minoru Ohsawa, Tetsuharu Kamiya, Tadashi Ishii.
